# Romania, a Harbour of HIV-1 Subtype F1: Where Are We after 33 Years of HIV-1 Infection?

**DOI:** 10.3390/v14092081

**Published:** 2022-09-19

**Authors:** Mădălina Preda, Loredana Cornelia Sabina Manolescu

**Affiliations:** 1 Department of Microbiology, Parasitology and Virology, Faculty of Midwives and Nursing, “Carol Davila” University of Medicine and Pharmacy, 020021 Bucharest, Romania; 2 Research Department, Marius Nasta Institute of Pneumology, 050159 Bucharest, Romania; 3 Department of Virology, Institute of Virology “Stefan S. Nicolau”, 030304 Bucharest, Romania

**Keywords:** subtype F1, HIV/AIDS, HIV-1 infection in Romania

## Abstract

Infection with the human immunodeficiency virus (HIV) has been a major public health concern worldwide for more than 30 years, including in Romania. The F1 HIV-1 subtype was exported from Angola to Romania most probably because of the two countries’ close political connections. Patients infected with HIV-1 via re-used and improperly sterilized injection equipment and through transfusions of unscreened blood, also known as the “Romanian cohort”, were the most common type of HIV-1 infection in Romania in the early 1990s, when the virus’s presence was recognized. Recently, subtype B started to increase in our country, mostly diagnosed in people using intravenous drugs or in men having sex with men. The evolution of the HIV-1 infection in Romania has been unique, with a dominance of the subtype F1, making it different from other countries in Europe.

## 1. Introduction

Infection with the human immunodeficiency virus (HIV-1) has been, for more than 30 years, a major public health concern around the world, including in Romania [1]. HIV-1 can be transmitted sexually (vaginal, pre-seminal, semen, or rectal fluids), through blood products, by sharing injecting drug products (injecting drug users (IDU)), or from mother-to-child [1].

Patients infected via re-use and improperly sterilized injection equipment and through transfusions of unscreened blood, often known as the “Romanian cohort”, were the most common type of HIV infection in Romania [1] in the early ninety’s, when the presence of the virus was acknowledged in our country. They were infected in the late 1980s and early 1990s (>90 percent in the early cases, under the age of 13 at the time of diagnosis) [1]. Most HIV-1-positive patients in Romania have HIV-1 subtype F, with a minor percentage of cases being IDU [1].

HIV-1 subtype F was first described in Romania in 1994, using samples from HIV-1 Romanian and Brazilian infected people [2]. Following a review of the African genomes, this subtype was reclassified into sub-subtypes F1 and F2 [3].

Despite its low overall incidence, subtype F1 is widely distributed, with high prevalence in some Central African, South American, and some European countries [4]. Until now, the majority of HIV-1 subtype F1 infections in Africa have been identified in Angola and the Democratic Republic of Congo (DRC) [4]. Angola, on the one hand, has a significant incidence of subtype F1 infections, ranging from 8% to 23%, making it one of the most common clades in the country, according to molecular epidemiology data [4]. The subtype F1 clade, on the other hand, accounts for only 5% of the HIV-1 strains circulating in the DRC [4]. Brazil has a significant incidence of subtype F1 and BF1 recombinant variants (10–20%) in South America [4].

Other Southern cone countries have a high prevalence of BF1 recombinants as well, but only occasional occurrences of “pure” subtype F1 have been reported [4]. The majority of subtype F1 infections in Europe are centered in Romania, where this subtype has a frequency of more than 70% [4].

## 2. HIV-1 Epidemiology in Romania

Because of the close political ties between the two countries, the F1 variant was exported from Angola to Romania (about 1960) [5,6]. Probably due to the political regime in the 1980s, the spread of HIV-1 resumed only at the subtype F1, no other variants were available because of reduced foreign connections with other countries. Subtype F1 appears to have spread in Romania via the heterosexual route among adults and the parenteral route among institutionalized youngsters [4].

In 1989, the first pediatric case of HIV-1 was recognized and recorded in Romania, followed by hundreds of cases in the following years [4,5], this fact suggesting the hidden HIV-1 infection at the beginning of the HIV-1 epidemic. Most Romanian children were horizontally infected by subtype F1 virus from the adult population, which likely entered a health care environment and was then disseminated using contaminated needles and syringes and/or transfusions of unscreened blood or blood products, according to phylogenetic and epidemiologic evidence [7,8].

In recent years, newly diagnosed patients have been young adults (24–35 years of age) that were infected through heterosexual contact [9]. Subtype B strains had previously been found to be on the rise, rising from 0% of all genotyped strains in 2003 to 7.8% in 2007 [9]. This variant is the second most common among newly diagnosed HIV-1 patients, behind subtype C and a number of CRFs and URFs [9] that also have been found.

In one Romanian study, men having sex with men (MSM) transmission was the most common risk factor found for subtype B, followed by heterosexual transmission [9]. MSM transmission was detected in 10.4% of patients in 2010, according to epidemiological statistics [9]. Although not particularly high, this figure is part of an upward trend (7.9% and only 1.8 percent of new HIV-1 cases reported in 2009 and 2006, respectively), possibly reflecting a reduced reluctance to disclose their sexual orientation in later years; overall, this sexual behavior risk factor is linked to only 1.78% of Romania’s HIV-1/AIDS cases [9]. MSM are five times more likely to carry a subtype B strain, than either a subtype F1 or C strain [9].

According to the last available report regarding the situation of HIV-1 infections in Romania, between 1985 and June 2022, there were a total of 26.554 cases of HIV-1/AIDS [10]. In 2021, there were 559 new cases reported in Romania, of which 76% were males [10].

**Figure 1:**
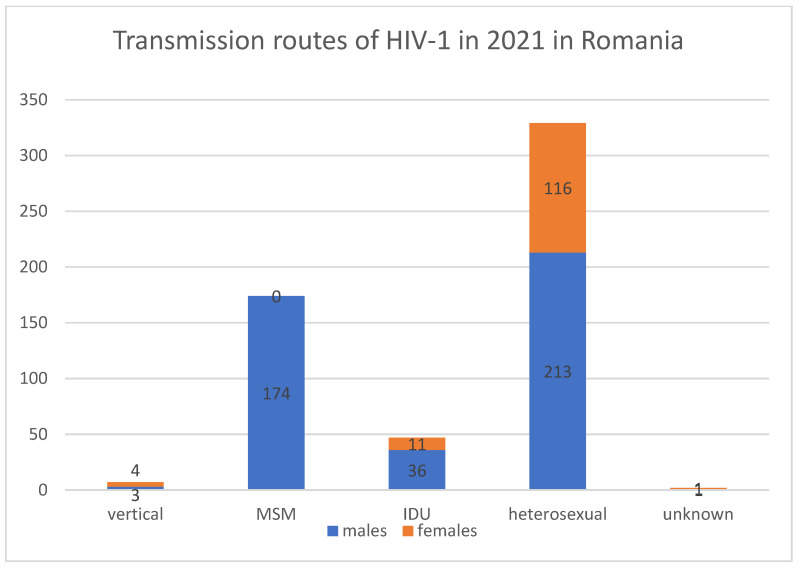
Transmission routes of HIV-1, in 2021, in Romania.

In 2021, the most frequent transmission route was through heterosexual transmission (Figure 1).

## 3. Subtypes of HIV-1

HIV is an enveloped, single stranded, positive sense RNA virus [11].

The reverse transcriptase of HIV-1 lacks a proofreading function; thus, it can lead to a high number of mutations during replication [12]. HIV recombination happens when a person is infected with two different strains of the virus that replicate within the same cell to yield recombinant viruses, which can result in increased genetic variation [12].

The virus is divided into two types: HIV-1 and HIV-2. The most common strain of HIV is HIV-1, which originated in the Congo Basin region of Africa. It accounts for approximately 96% of all HIV infections [13].

HIV-1 has a high degree of genetic variation and has been divided into groups, subtypes, and sub-subtypes based on phylogenetic clustering. Subtypes and sub-subtypes originate from a post-introduction founder event and continue diversification, whereas groups correspond to discrete lineages independently brought into the human population from non-human primates. These distinctions have resulted in the formal identification of four groups (M, N, O, and P) [14].

Group M can be split into nine subtypes (A–D, F–H, J, and K) based on genetic variation, with intra- and inter-subtype variances of 17–35% and 8–17%, respectively, depending on the genomic area examined [13], and several sub-subtypes (A1, A2 for subtype A and F1, F2 for subtype F) [14].

The largest diversity of subtypes and recombinant forms has been found in Central Africa [13]. This region contains all of the subtypes as well as many recombinant forms [13]. CRF02 AG (one of the circulating recombinant versions) (46%) and subtype G have been found in considerable proportions in west Africa (27%) [13]. Subtype A was responsible for over half of all infections in east Africa, with considerable numbers also coming from subtypes C, D, and URFs [13].

Subtype A was responsible for more than half of the infections in Eastern Europe and Central Asia, with subtype B and CRFs also present [15,16].

Repeated transmission of the HIV-1 variant A_FSU_, now identified as a unique subtype A sub-subtype termed A6, was a hallmark of HIV-1 outbreaks in the nations of the Former Soviet Union (FSU) [17]. Sequences from numerous non-FSU nations, including Bulgaria, Cyprus, the Czech Republic, Mongolia, Poland, Slovenia, and Turkey, were also present in the A6 cluster [17]. Except for the Czech Republic, where A6 sequences date from 1998 to 2000, most of the A6 sequences from non-FSU countries were discovered more recently, having been deposited between 2002 and 2017 [17].

Given the tremendous genetic diversity of HIV-1, panels of pseudoviruses need to be updated often to reflect the development of new strains. Thirteen HIV-1 env-pseudoviruses of the recombinant form CRF63 02A and subtype A6 were obtained based on genetic variations of HIV-1 circulating in the territories of the Siberian Federal District [18].

Subtype B infection rates were high across Western and Central Europe, North America, the Caribbean, Latin America, and Oceania, accounting for at least 75% of infections [13].

In Romania, the HIV-1 epidemic began with subtype F1, which curiously remained the most common, although the epidemic has evolved over time [9]. This is an interesting epidemiological trait, since in other neighboring countries of the former USSR the main subtype is A, followed by subtype B, the second most common variant, CRF03_AB, CRF02_AG and CRF02_AG [15,19].

HIV-1 subtype B phylogenetic research has revealed two separate pathways of propagation [9]. Paraschiv and colleagues discovered a single big MSM Bucharest-specific cluster, implying that infection among this population’s individuals was spread by a single founder event [9]. The remaining virus isolates were similar with reference strains from other European countries, mostly from the west of the continent, to produce single branches or extremely tiny clades [9]. This trend shows that single introductions were occasionally followed by limited diffusion, which was consistent with the patient’s reported illness source [9].

In the Middle East and North Africa, the number of infections caused by subtype B has dropped, while the proportion of CRFs (mostly CRF35 AD) has increased [13,19].

In Southern Africa and India, subtype C is most prevalent, accounting for 99% and 95% of infections, respectively [13]. Subtype C infections account for about half of all HIV-1 infections worldwide (47%) [13]. The circulating recombinant forms CRF02 AG (8%) and CRF01 AE (8%) are responsible for 12% and 10% of infections, respectively (5%) [13].

More than ten BF1 mosaics display 11 distinct recombination profiles: six are singleton unique-recombinant-forms/URFs, one exhibits a CRF28/29 BF-like recombinant pattern, and the other four BF1 isolates branch with other Brazilian BF1 viruses previously described and may represent putative new CRF BF1 from Northern Brazil. Blood donors from Brazil exhibit a relatively uniform molecular pattern with subtypes B and BF1 being the most common, followed by subtype C and F1 on a random basis [20]. In a highly endemic, distant, and physically isolated location as Northern Brazil, surveillance studies are vital to monitor HIV-1 variety which can indicate patterns of viral spread [20].

Phylogenetic analyses have revealed that the Romanian C strains were quite diverse, clustering into many groups defined by a shared transmission method (transfusion/surgical procedures) or limited geographic relatedness [21]. In Romania, the HIV-1 epidemics for subtypes F and C have appeared to follow separate patterns of transmission [21]. While subtype F1 appeared to have been monoclonally introduced and widely distributed in the 1980s, subtype C strains, while present in the late 1980s, did not spread as widely [21].

CRFs (particularly CRF01 AE) are the most common infections in Southeast Asia and East Asia. At 80%, the two regions have the largest proportion of recombinant forms in the world [13].

## 4. Particularities of HIV-1 Subtype F

### 4.1. Origin of HIV-1 and Spread of HIV-1 F1

Phylogeographic analyses of the pandemic branch of HIV-1 group M, have indicated that this clade originated in western-central Africa around 1900–1930 [4]. The virus is thought to have propagated among people along the Congo River to Kinshasa, Zaire, where the first documented instance of HIV-1 infection (group M strain) in humans was linked to a blood sample from 1959 [12].

The F1 sub-subtype has been found in European (Romania, Italy, Spain, and Russia) and South American (Argentina, Chile, Bolivia, and Brazil) countries, while the F2 sub-subtype is predominantly found in Central African countries [3].

The epidemiological environment in which the subtype F1 spread occurred differs dramatically among countries [4]. In Angola, the major epidemic driving force was heterosexual intercourse and the country’s long-running civil conflict, which may have had a significant impact on the HIV-1 pandemic’s growth pattern [4,7]. Subtype F1 was found among heterosexual, gay, and intravenous drug users (IDU) in Brazil, suggesting that both sexual and iatrogenic routes may have played a role in viral transmission [4].

### 4.2. HIV-1 F1 in Romania and Global Context

The prevalence of HIV-1 sub-subtype F1 has been estimated to be 0.45% worldwide, based on data collected between 2000 and 2007 [3]. Only 58 (0.8%) of the 6948 full HIV-1 genomic sequences in the Los Alamos HIV-1 database belong to the F1 sub-subtype, with 15 (0.2%) from Brazil [3]. Only one CRF BF1 was found outside of South America, and nine of the fifteen CRF BF1 were found in Brazilians [3]. Founder effect, host restriction factors, and cultural/behavioral factors may all play important roles in the unequal global dissemination of HIV-1 variants in humans [22].

Several investigations have indicated that the South American and Romanian epidemics were caused by separate subtype F1 introductions; yet, the geographic centers of these subtype F1 epidemics have been one of the most perplexing features of HIV-1′s global propagation [23].

**Figure 2:**
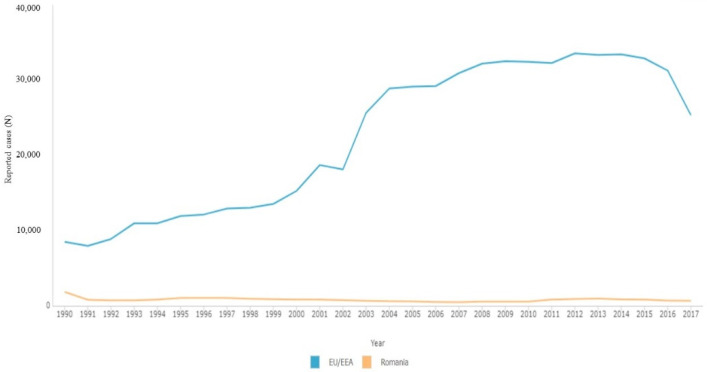
Evolution of the number of cases in Romania as compared with Europe (image created using the Surveillance Atlas of Infectious Diseases, ECDC [25]).

After an initial period of exponential growth that lasted until the early 1990s, the parenteral epidemic has generally maintained a stable population size (Figure 2) [24]. This shows that, since the early 1990s, there have been very few parenteral transmissions and subsequent virus propagation within this group [24]. This can be explained by the fact that the infections occurred in youngsters who were unlikely to participate in high-risk activity, and that public health initiatives to stop the epidemic from spreading, which began in the early 1990s, were a huge success [24]. A phylogeographic study revealed that the adult population in Bucharest, Constanța, and Giurgiu was the epicenter of the outbreak in the early 1980s [24]. However, it is likely that these sites were not the ultimate source of the parenteral pandemic, because the children could have moved throughout Romania or the world since contracting the virus in 1989, when the Communist state fell [24]. This could explain why the monophyletic cluster from the United States, Germany, and Austria contains three non-Romanian sequences [24].

Subtype B and F strains, as well as recombinants of both subtypes B and F, have been seen in South American HIV-1 outbreaks [26]. In some places, the prevalence of BF recombinants was comparable to that of subtype B strains, which was much higher than that of pure subtype F strains [26].

Brazil and Romania were probably both responsible for the outbreak of the F1 virus in Italy, but Brazilian strains contributed to many more introduction episodes with a wider spread [27]. Since then, the prevalence of sub-subtype F1 has steadily increased among Italian descendants, primarily among self-declared heterosexual men, despite phylogenetic analyses suggesting that a few of these self-declared heterosexual men may have contracted the virus through homosexual interaction [27].

**Figure 3:**
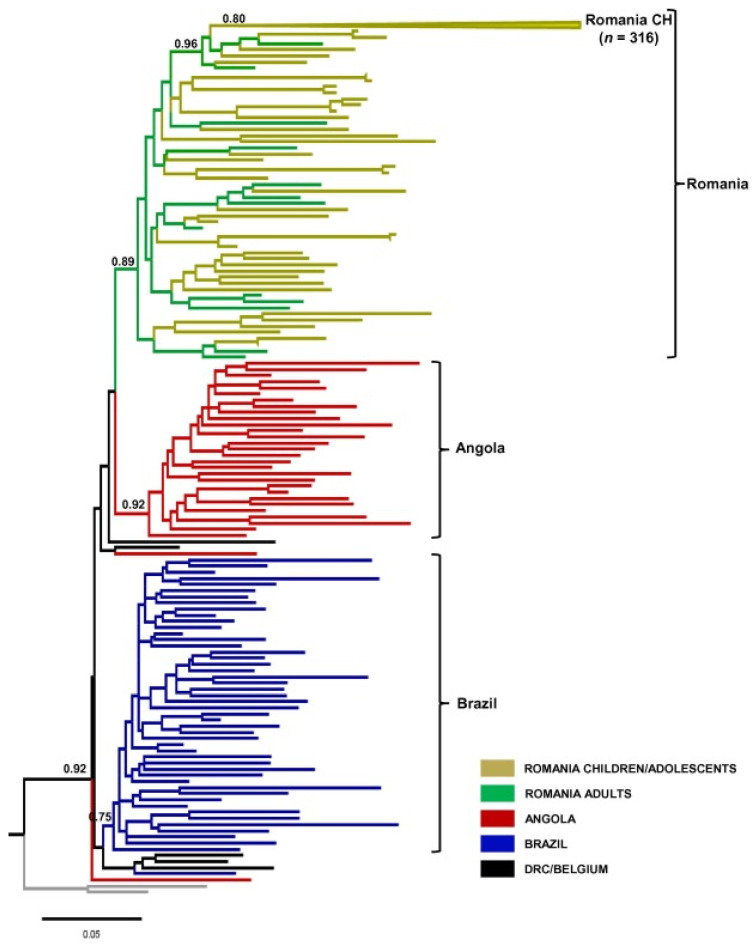
Phylogenetic analysis of the *pol* gene of HIV-1 subtype F1 strains circulating in Angola, Brazil, Romania, the DRC, and Belgium (linked to the DRC) (image source: [4]).

Phylogenetic investigations of subtype F1 strains identified around the world have revealed that subtype F1 first diverged in the DRC, and then moved to Angola, Romania, and South America (Figure 3) [4,23].

Most sequences from Angola, Brazil, and Romania segregated into nationwide phylogenetically unique groups, whereas most subtype F1 sequences from Romanian children branched as a monophyletic subcluster nested within sequences from adults, according to phylogenetic analyses of HIV-1 subtype F1 pol sequences sampled worldwide [4].

The estimate periods of the most recent common ancestors of the different subtype F1 clades were Angola = 1983 (1978–1989), Brazil = 1977 (1972–1981), Romania adults = 1980 (1973–1987), and Romania children = 1985 [4].

A model of logistic population increases best explained the demographic history of all subtype F1 clades [4]. Although the expansion phase of the subtype F1 epidemic in Angola (mid-1980s to early 2000s) coincided with the country’s civil war (1975–2002), on the one hand, the average estimated growth rate of the Angolan F1 clade (0.49 year (−1)) was not particularly high but was comparable to the Brazilian (0.69 year (−1)) and Romanian adult (0.36 year (−1)) subtype F1 clades [4]. The Romanian children subtype F1 lineage, on the other hand, had a rapid and short dissemination phase, with a median growth rate (2.47 year (−1) that was significantly higher than that of adult populations [4].

Although prior research has suggested that another sub-subtype F1 lineage traveled from Brazil to Europe in the 1990s, this lineage only recently reached epidemic levels in two nations, Belgium and Spain, about 16 and 12 years ago, respectively [27]. F1 was introduced into local MSM networks in both cases, which was most likely a factor in its rapid spread and circulation [27].

## 5. Evolution of HIV-1 in Romania

In Romania, HIV-1 isolates obtained from adolescents and adults who were infected as children formed a unique category [28]. Resistance-associated polymorphisms in the protease gene were distributed differentially in the teenage and older groups, i.e., more mutations have been described in adult patient strains than in adolescent patient strains [28].

In both IDUs and sexually infected individuals, HIV-1 subtype analyses revealed a predominance of the local F1 strain; in IDUs, it also revealed 28 CRF14_BG recombinants and 6 URFs between F1 and CRF14_BG [29]. Subtype B infection was found in a few patients from both risk groups [29]. CRF14 BG was linked to a lower CD4 cell count and more advanced illness stages in IDUs, which was linked to CXCR4 tropism [29]. A phylogenetic study revealed that HIV-1 has spread recently through three large IDU clusters. Some CRF14 BG IDUs have been reported traveling abroad (Spain, Greece) [29]. Molecular epidemiology approaches have provided vital information on HIV-1 transmission patterns by detecting clusters of IDUs with related infections [29]. This knowledge can be used to develop relevant harm reduction programs [29]. The recent rapid increase in the frequency of injectable drug-related HIV-1 transmissions in Romania has resulted in close-knit transmission networks and diversification of circulating subtypes, marking a significant shift in the genetic profile of the Romanian HIV-1 epidemic [30].

The majority of therapeutic experiences have come from HIV-1 subtype B infections; treatment of patients infected with HIV-1 strains other than B is most often an extrapolation [28].

In subtype F isolates obtained from Romanian patients, no significant mutations linked with resistance to NRTIs, NNRTIs, or PIs have been reported, but many accessory substitutions have been discovered as spontaneous variants, especially in places responsible for resistance to protease inhibitors [28]. Their prevalence differs slightly from that of other subtype F HIV-1 strains [28]. All Romanian strains had the M36I mutation in the protease gene, which was linked to resistance to ritonavir and nelfinavir in B subtype viruses [28].

TAMs (thymidine analog mutations) and M184V were the most prevalent mutations [31]. Transmitted HIV-1 drug resistance (TDR) was 14.75%, mostly associated with NRTI resistance (13.11%). TDR fell from 26.08% in 1997–2004 to 7.89% in 2005–2011, indicating a downward trend [31].

Recent seroconverters have shown no signs of initial resistance [31]. Minor changes in the protease and RT genes were found in all HIV-1 strains, typically in polymorphic locations [31].

The lowering incidence of TDR might be related to the strong efficacy of HAART and to the rising number of treated patients with virological success who have a low risk of transmission [31]. The rapid rise in HIV-1 infections including other subtypes necessitates ongoing monitoring of the epidemic’s genetic composition [31]. It is important to recall that Romanian HIV-1 infected patients benefited from the beginning of the HIV-1 epidemic of all existing HIV-1 drugs.

Isolates from long-term survivors of HIV-1 infection who were parenterally infected as children and had many virologic treatment failures were genotyped for treatment resistance and subtype identification in a Romanian study [32]; 94.7 percent of the patients had subtype F1 strains, which clustered with other Romanian and Angolan F1 strains [32]. Although sequences from the DRC fell in a basal position within the subtype F1 phylogeny, they were only weakly connected with the South American and Romanian clades, according to the most comprehensive phylogenetic analysis of subtype F1 strains circulating worldwide to date [23]. Despite the lengthy and complicated treatments, 15.8% of patients had wild type virus, 68.4% were fully susceptible to protease inhibitors, 47.3 percent to non-nucleoside reverstranscriptase inhibitors, and 28.9% to nucleoside reverstranscriptase inhibitors, and 28.9% to nucleoside reverstranscriptase inhibitors [32]. Only 13.2% of people tested positive for resistance to all antiretroviral medication classes [32]. Severely immunosuppressed patients had a significantly higher total number of mutations, as well as major mutations in the protease gene (V82A, I54V, G48V) and the major M184V mutation associated with type 2 thymidine analogue mutations in the reverstranscriptase gene [32,33].

## 6. Conclusions

HIV-1 infection in Romania is unique both through the increased percentage of HIV-1 subtype F, more frequent than other European countries, but also by its infected cohort of children before 1989. Thanks to the genetic studies performed, the origin of the strains from our country has been established as Angola, and the time estimated to the most common ancestor has been established as 1983 for adults. Most of the studies have been targeted on the subtype B, thus the majority of data regarding the evolution and treatment of HIV-1 was extrapolated to subtype F. Recently, more and more studies that have included strains from our country have been published; in the 2010s, an increase in the number of HIV-1 subtype B cases, near the capital city, especially in IDU, has been noticed. The main type of transmission in Romania, at the moment, remains to be heterosexual transmission, followed by MSM.

## Data Availability

Not applicable.
